# Alzheimer's disease and insomnia: a bibliometric study and visualization analysis

**DOI:** 10.3389/fnagi.2025.1542607

**Published:** 2025-04-08

**Authors:** Zi-Yue Tian, Bing Jiang, Meng Jin, Xiao-Kun Yu, Qi-Lin Chen, Jia-Hui Wang

**Affiliations:** ^1^Hainan General Hospital, Hainan Affiliated Hospital of Hainan Medical University, Haikou, Hainan, China; ^2^Department of Integrated Chinese and Western Medicine, Gansu University of Chinese Medicine, Lanzhou, Gansu, China; ^3^The Third Affiliated Hospital of Changchun University of Traditional Chinese Medicine, Changchun, Jilin, China; ^4^The Affiliated Hospital of Changchun University of Traditional Chinese Medicine, Changchun, Jilin, China; ^5^KweiChow Moutai Hospital, Zunyi, Guizhou, China

**Keywords:** bibliometrics, CiteSpace, VOSviewer, visual analysis, insomnia, Alzheimer's disease

## Abstract

**Background:**

Alzheimer's disease (AD) is the fastest-growing neurodegenerative disorder globally, with patient numbers expected to rise to 130 million by 2050. Insomnia, a prevalent comorbidity, exhibits a bidirectional relationship with AD: insomnia accelerates AD pathology, while AD worsens sleep disorders. This relationship has emerged as a key area of research. Current mechanisms involve oxidative stress, inflammatory responses, and glymphatic system dysfunction, yet a comprehensive review of these processes remains absent.

**Objective:**

To conduct a visual analysis of the relationship between Alzheimer's disease and insomnia using CiteSpace.

**Methods:**

Literature on “insomnia” and “Alzheimer's disease” published between January 1, 2000, and October 31, 2024, was retrieved from the Web of Science Core Collection. CiteSpace and VOSviewer software were used to analyze institutions, authors, and keywords.

**Results:**

A total of 1,907 articles were analyzed, revealing a consistent upward trend in publication volume. The United States and the Mayo Clinic were identified as leading contributors, producing 704 and 57 publications, respectively. Boeve Bradley F the most prolific author contributed 30 publications. Collaboration was actively observed among countries, institutions, and authors. High-frequency keywords identified were “Parkinson's disease,” “cognitive impairment,” and “sleep behavior disorder.” Emerging research areas are likely to focus on “sleep quality” and the “glymphatic system.”

**Conclusion:**

This study is the first to apply bibliometric analysis to identify three key trends in AD and insomnia research: the dominance of the United States and Mayo Clinic, strong international collaboration, and a focus on critical areas such as cognitive impairment, the glymphatic system, and sleep interventions. Insomnia may accelerate AD progression via multiple pathways, indicating that enhancing sleep quality could provide new strategies for early intervention. Future research should prioritize advancing the clinical translation of sleep interventions and investigating the mechanisms of the glymphatic system.

## 1 Introduction

Alzheimer's disease (AD) is a neurodegenerative disorder marked by progressive cognitive decline, memory loss, and behavioral changes, which significantly affect patients' daily lives and social interactions (Li et al., 2023). The prevalence of AD is increasing rapidly among aging populations and is expected to nearly triple in the next few decades (Wilkins et al., 2022). AD imposes substantial, psychological, and financial burdens on patients and their families and places significant strain on healthcare systems.

Insomnia, a prevalent sleep disorder, is strongly linked to the onset and progression of Alzheimer's disease. Studies show that AD patients frequently suffer from poor sleep quality, including difficulty falling asleep, frequent nighttime awakenings, and excessive daytime sleepiness (Webster et al., 2019). Such disturbances may result from neurotransmitter imbalances in AD, including norepinephrine and acetylcholine dysregulation, which disrupt the sleep-wake cycle (Huang et al., 2020). Insomnia impairs cognitive function and may also accelerate disease progression, perpetuating a vicious cycle (Musiek and Holtzman, 2016). Improving sleep quality is therefore essential for the overall wellbeing of AD patients.

Current treatments for AD mainly involve medications, such as donepezil, rivastigmine, and memantine, aimed at improving cognitive function (Khan et al., 2022). However, these drugs show limited effectiveness in improving sleep quality and may cause adverse effects with prolonged use, such as gastrointestinal issues, nausea, and even insomnia (Arvanitakis et al., 2019). Combining pharmacological treatments with non-pharmacological interventions, such as cognitive behavioral therapy (CBT) and sleep hygiene education, is crucial for improving sleep quality in AD patients (Qaseem et al., 2016).

The interaction between sleep and circadian rhythms plays a critical role in Alzheimer's disease. Circadian rhythm disruptions can worsen insomnia and further impair cognitive function. Borbély's “two-process model” provides a framework for understanding sleep regulation in AD, emphasizing the interaction between circadian rhythms and sleep homeostasis (Borbély, 1982). Addressing sleep disturbances and circadian rhythm dysregulation in AD may offer novel clinical intervention strategies to enhance cognitive function and quality of life.

Bibliometrics is a quantitative method used to systematically analyze academic literature (such as books, journal articles) and associated metadata (such as abstracts, keywords, and citations). Bibliometric analysis maps relationships between publications and identifies trends in specific disciplines or research fields by analyzing statistical data. Despite its limitations, bibliometrics is a widely used objective tool for understanding various aspects of research activity (Chen, 2006).

This study applied bibliometric and visual analyses with VOSviewer and CiteSpace to explore the relationship between AD and insomnia. The analysis quantitatively evaluated contributions from authors, countries, and institutions, along with their collaborative networks (Synnestvedt et al., 2005; van Eck and Waltman, 2010). Bibliometric tools were also employed to analyze keywords in the included literature, identifying research hotspots and forecasting future trends. These findings offer valuable insights for researchers aiming to understand the current state of the field.

## 2 Methods

### 2.1 Data sources and search strategy

This study's terminology is based on a frequency analysis of high-frequency terms from the MeSH database, focusing on core concepts. Insomnia is defined by the ICSD-3 criteria as difficulty falling asleep, maintaining sleep, or early awakening for at least 3 months, along with daytime functional impairment. The selected search terms include “Insomnia,” “Sleep disorder,” “Sleeplessness,” and “Circadian rhythm disruption,” while non-specific terms (e.g., “fatigue”) were excluded to ensure relevance. Articles on Alzheimer's disease and insomnia were retrieved from the Web of Science Core Collection (WoSCC) between 2000 and 2024. Retrieval strategy: [TS = (“Alzheimer's disease” OR “Alzheimer's disease”) AND TS = (“Insomnia” OR “Sleep disorder” OR “Sleeplessness” OR “Circadian rhythm disruption”)] NOT TS = (“Parkinson's^*^”); Publication Date: 2000-01-01 to 2024-10-31. A total of 2,049 articles were retrieved.

### 2.2 Study type and exclusion criteria

**Study type**: This includes both original research articles and reviews, which together provide a comprehensive overview of the existing knowledge.

**Exclusion criteria**: Language: Only studies published in English were included to ensure consistency and reliability. Relevance: Studies not directly addressing the relationship between Alzheimer's disease and insomnia were excluded. This includes studies that focus on only one condition without exploring their potential connection.

Quality: Studies with poor methodological quality or that did not meet the standards of peer-reviewed journals were excluded to maintain the analysis's integrity.

**Figure 1:**
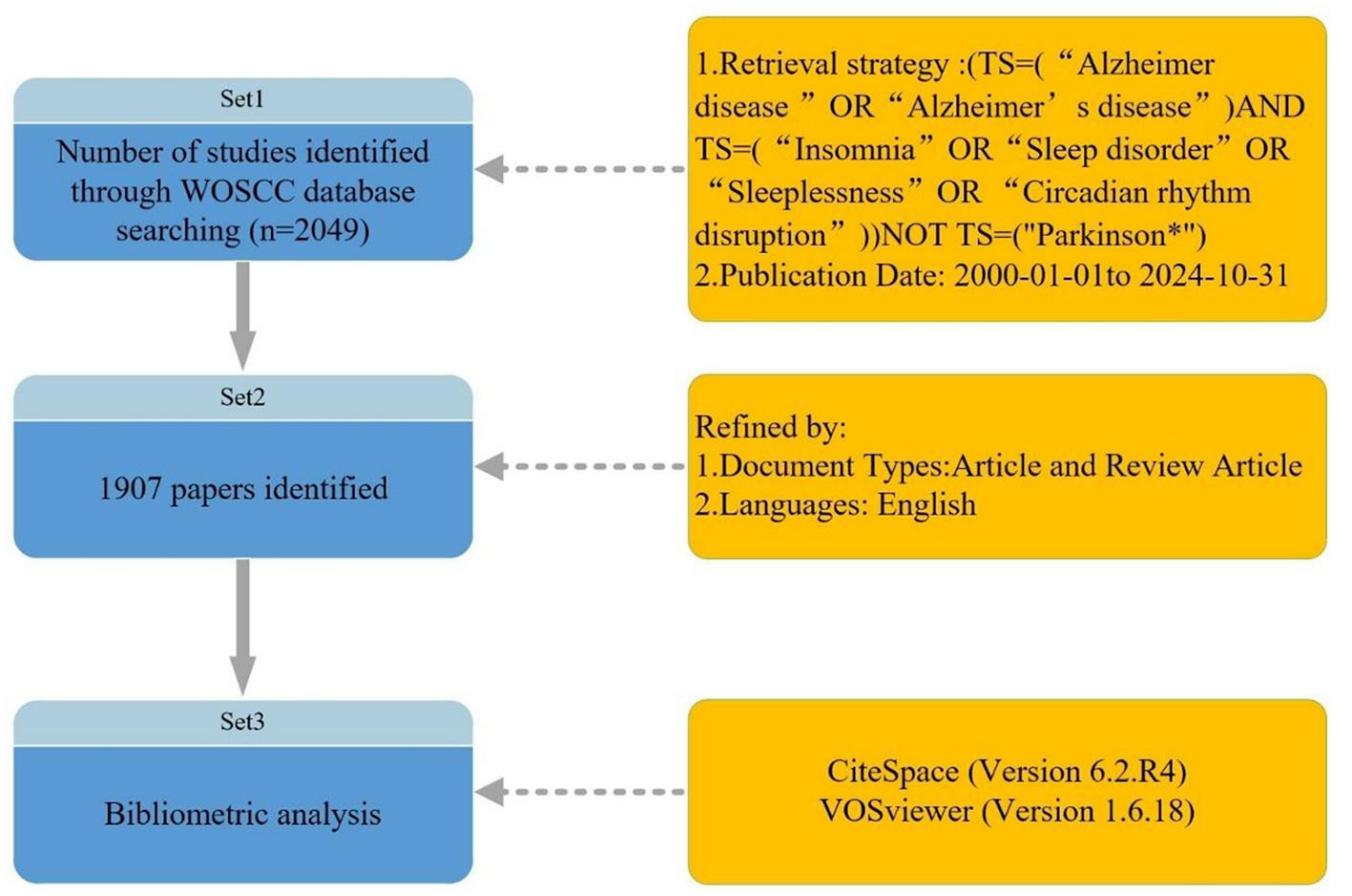
Flowchart of data screening and bibliometric analysis.

**Figure 2:**
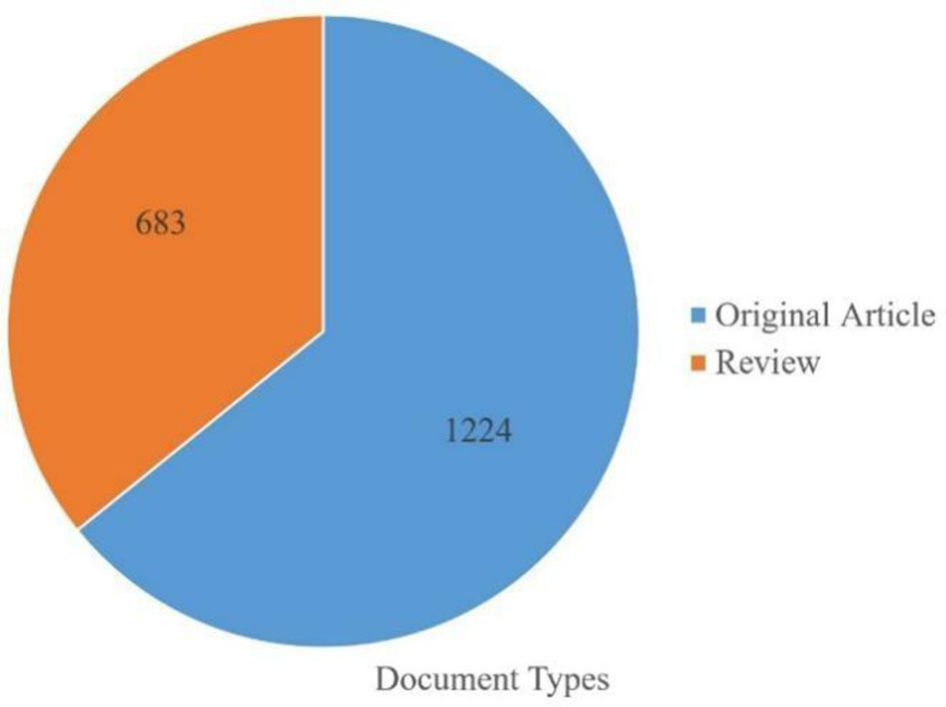
Distribution of documents by type.

After applying these steps, 1,907 articles were selected, and plain text files were used to export complete records and references. The extracted data were downloaded into CiteSpace and VOSviewer software for bibliometric analysis. The search strategy employed in this study is shown in Figure 1. The literature mainly consists of original articles (1,224 in total), with 683 reviews, as shown in Figure 2.

### 2.3 Visualization tools

This study used CiteSpace (version 6.2.R4) and VOSviewer (version 1.6.18) for co-occurrence analysis and to generate visualized graphs. The results of the analysis were imported into Microsoft Excel 2019 to produce supplementary charts and figures. In the visualized maps, nodes, and links constitute the core structure. Each node represents an analyzed entity, such as an author, institution, or journal. Links represent co-occurrence and co-citation relationships between nodes. Generally, a larger number of nodes indicates higher frequency, whereas the number and thickness of links reflect the strength of relationships between nodes. Centrality is a critical metric for assessing the importance of nodes within a network. Nodes with more shortest paths passing through them exhibit higher centrality. Nodes with centrality values >0.1 are generally considered pivotal within a specific field.

## 3 Results

### 3.1 Annual publication trends and analysis

**Figure 3:**
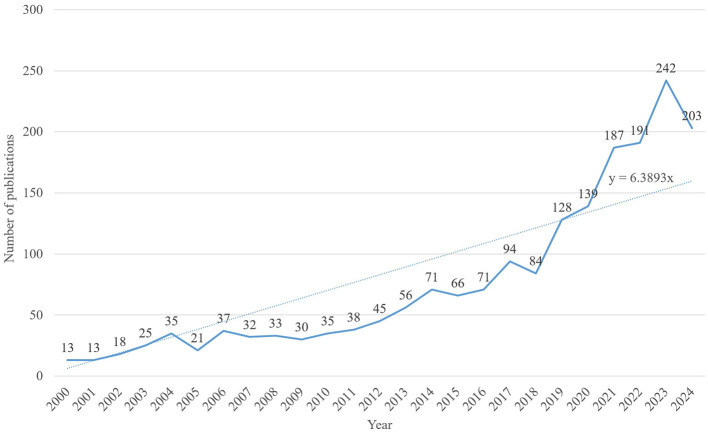
Annual publication trends. Shows the global publication volume from 2000 to 2024, with the fitted formula *y* = 6.3893*x*.

A literature search was conducted for studies published between 2000 and 2024. While early research laid the foundation for this field, it had limitations due to small sample sizes and lower research quality, which hindered the exploration of the complex mechanisms underlying AD pathology. Thus, focusing on high-quality research published after 2000 enables more accurate and reliable conclusions in this field. A total of 1,907 articles on AD and insomnia were retrieved between 2000 and 2024. As data collection ended on October 31, 2024, the records for 2024 are incomplete. Figure 3 illustrates a steady increase in publications from 2000 to 2023. The Joinpoint Regression model aims to identify “inflection points” or significant change points in the data, revealing trends in data changes by applying different regression models at various stages. In this study, we used this model to identify inflection points in publication volume between 2000 and 2023 (*p* < 0.05). A regression analysis of the annual number of articles published over the past 24 years yielded the linear growth formula: *y* = 6.3893*x*. This formula was derived using linear regression, a method that minimizes the distance (errors) between data points and the fitted line to determine the best-fitting line. The goal of this process is to use a mathematical model to describe the trend of data changes. The resulting linear growth trend indicates an annual increase of 6.4% in attention. Based on these “inflection points” or significant change points, the study divides the entire data period into four phases. The growth trajectory is divided into four phases: 2000–2005: A slow growth phase, with 20–35 articles published annually, representing the early stage of research interest. 2006–2011: A phase of modest fluctuations in research attention but with overall steady growth. 2012–2017: A steady growth phase, with publications increasing from 45 to 94, reflecting significantly heightened research interest. 2018–2023: A rapid growth phase, especially after 2020, characterized by a sharp surge in publications. The highest number of publications was recorded in 2023 (242 articles), with a slight decline to 203 articles in 2024. Overall, researchers' interest in the link between AD and insomnia has remained consistently strong. Annual publication trends underscore the growing importance of this academic topic. The steady rise in annual publications over the past 24 years demonstrates the increasing attention this topic has garnered from researchers.

### 3.2 Bibliometric analysis of countries and institutions

**Figure 4:**
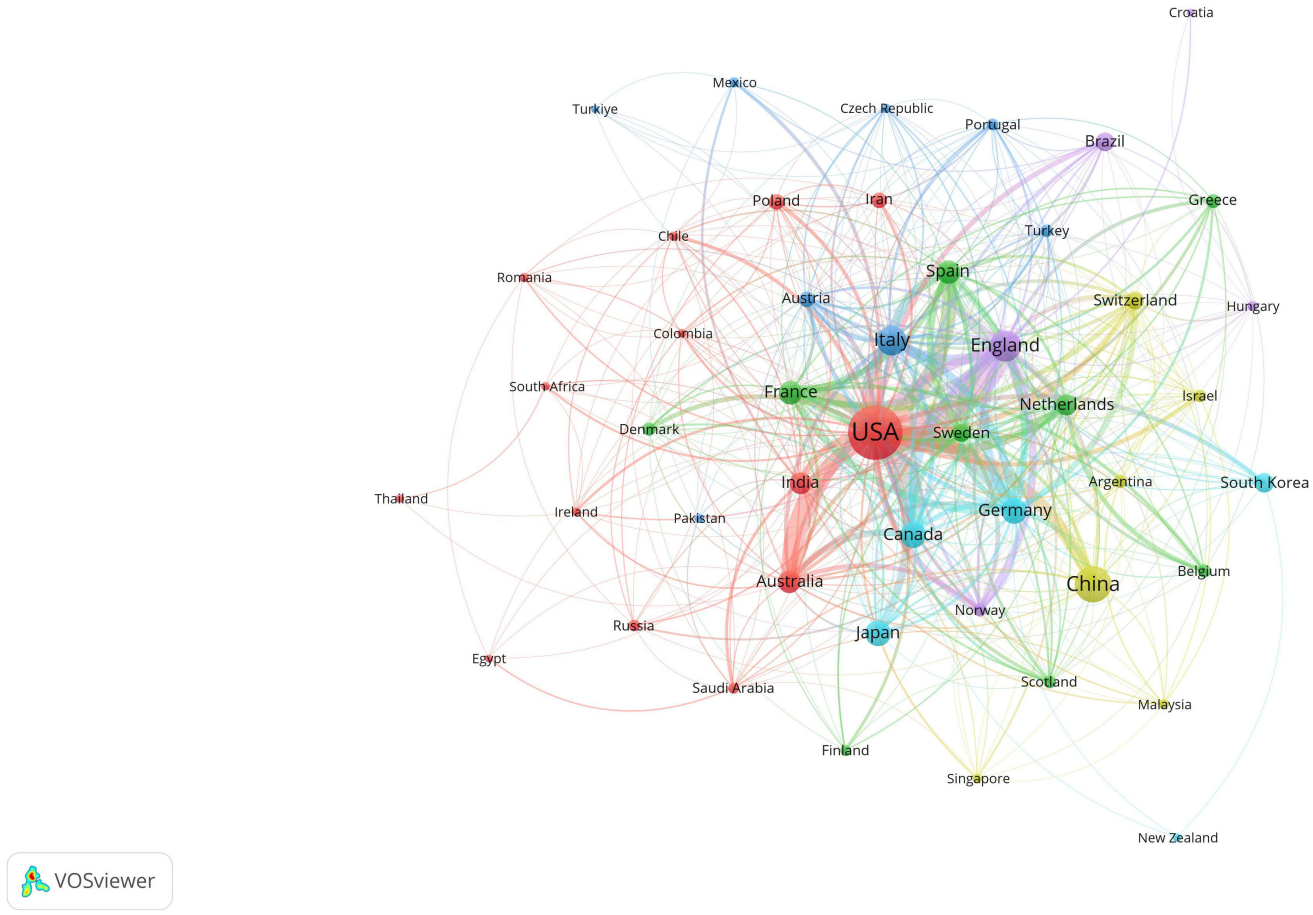
Collaboration network among countries and regions. The size of the nodes represents publication volume, while the thickness of the connecting lines indicates the strength of collaboration (TLS ≥ 10). Only 48 countries with publication volumes ≥5 are displayed. Six collaboration clusters, each represented by a different color, were formed, with a total connection strength of 1,753.

**Figure 5:**
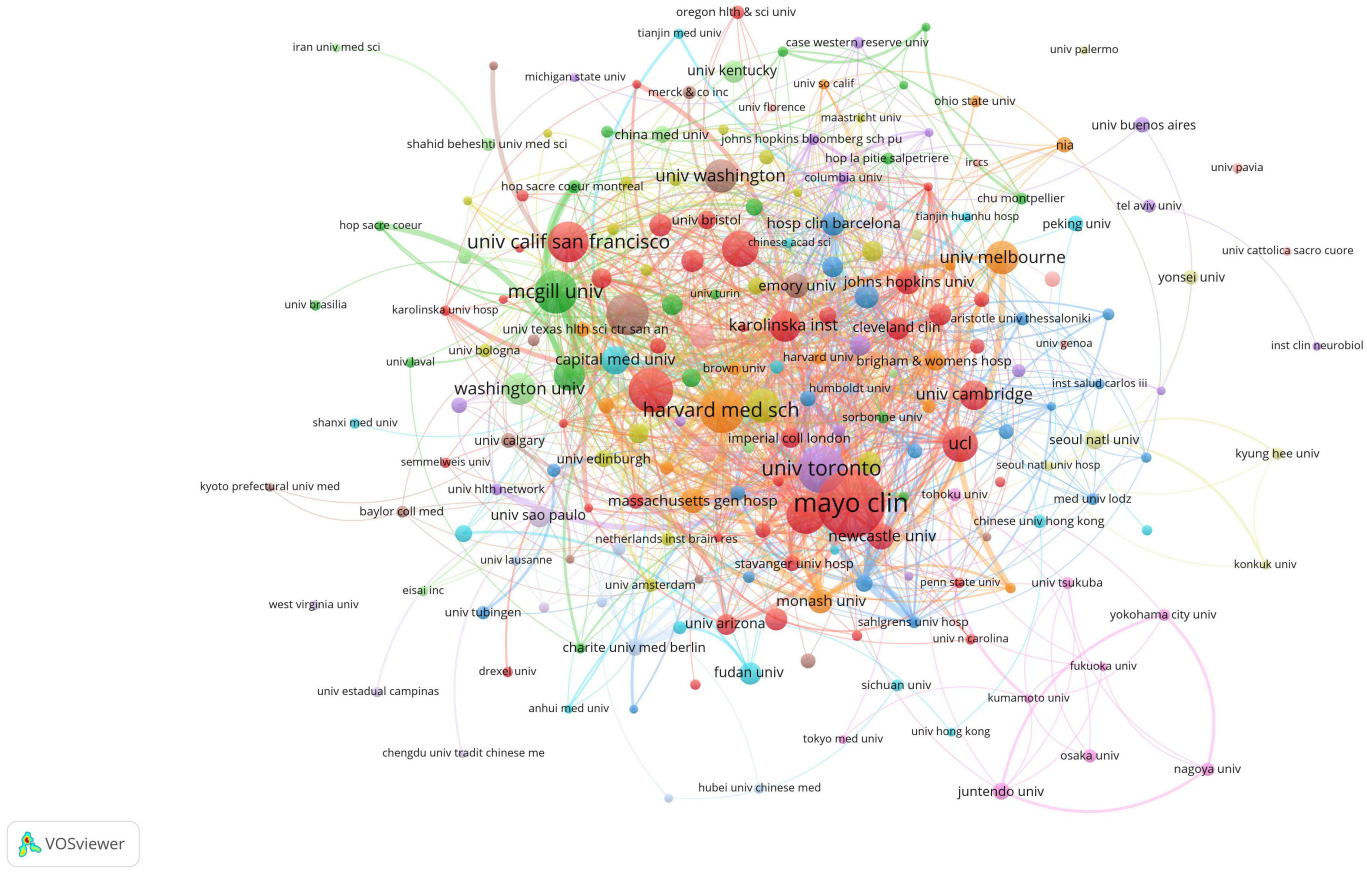
Institutional collaboration network. The size of the nodes represents publication volume, while the thickness of the connecting lines indicates collaboration strength (TLS ≥ 10). There are 2,862 institutions in total, with 216 institutions displaying a publication volume of 5 or more. Fourteen collaboration clusters, each represented by a different color, were formed, with a total connection strength of 1,913.

**Figure 6:**
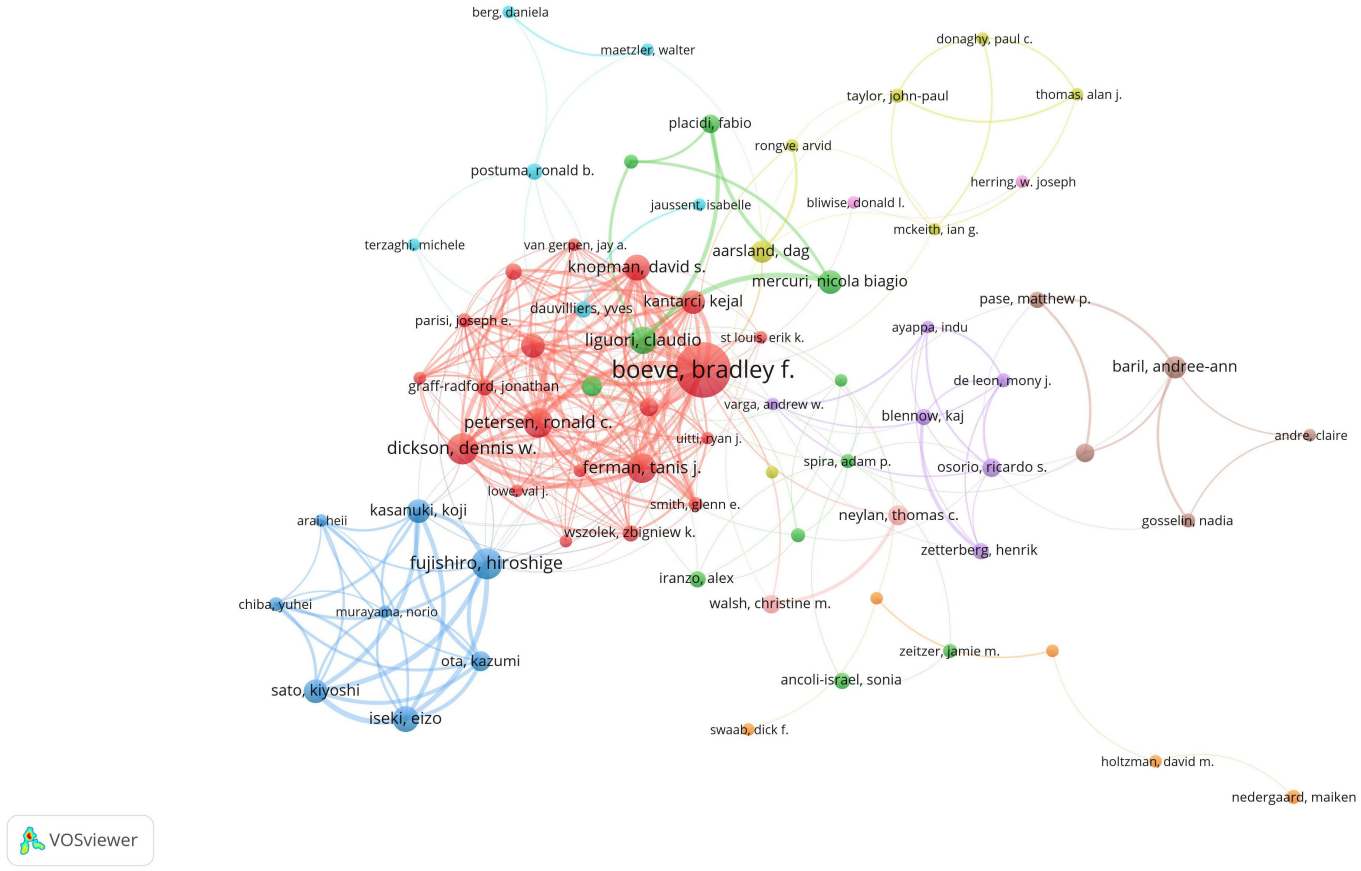
Author collaboration network. There are 9,560 authors in total. The size of the nodes represents publication volume, while the thickness of the connecting lines indicates collaboration strength (TLS ≥ 10). Only 96 authors with a publication volume of 5 or more are displayed. Eight collaboration clusters, each represented by a different color, were formed, with a total connection strength of 1,043.

Statistical analysis identified 2,862 institutions from 82 countries and regions that published articles on Alzheimer's disease and insomnia. The number of articles produced by each institution was analyzed using VOS viewer, as depicted in Figures 4–6.

Table 1 presents the top 10 contributing countries and regions. The United States led with 704 publications, followed by China with 267. The United Kingdom and Italy produced a comparable number of publications, with 179 and 159 articles, respectively. Figure 4 highlights the United States, United Kingdom, Germany, Italy, and Canada as prominent hubs with strong interconnections, indicating their significant global academic influence and collaboration in this field. Regarding collaboration, the United States ranked first with a Total Link Strength (TLS) of 500 and 44 connections, followed by the United Kingdom with a TLS of 307 and 37 connections.

**Table 1:** The top 10 most productive countries are ranked according to key metrics, including the number of documents, citations, total link strength (TLS), and the number of collaborative connections (Links).

**Rank**	**Country**	**Documents**	**Citations**	**Links**	**TLS**
1	USA	704	43,779	44	500
2	England	179	15,667	37	307
3	Germany	111	12,362	37	221
4	Italy	159	9,470	36	196
5	Canada	112	13,101	34	171
6	Spain	91	10,794	33	185
7	Australia	82	10,269	32	154
8	France	86	11,229	29	163
9	Peoples R China	267	7,689	25	97
10	Japan	114	9,134	25	68

Figure 5 shows 67 institutions that produced at least 10 publications. In North America, prominent institutions included the Mayo Clinic, Harvard Medical School, University of Pennsylvania, Stanford University, and University of California (San Francisco and San Diego), as well as Canada's University of Toronto and McGill University. In Europe, King's College London and University College London were significant contributors. These findings suggest that regional characteristics significantly influence institutional collaborations.

Table 2 highlights that the top ten global institutions in AD and insomnia research are dominated by North American institutions, with notable collaboration between Europe and North America. The Mayo Clinic in the United States leads with 57 publications and 3,680 total citations, focusing on the mechanisms of circadian rhythm disruption and the glymphatic system. The University of Toronto (37 publications) and McGill University (33 publications) in Canada investigate the interaction between the APOE ε4 gene and sleep disorders through extensive inter-institutional collaboration (49/50 links). King's College London (29 publications) leads Europe with a total link strength of 103, advancing multidisciplinary research on the socioeconomic burden of insomnia in AD. These institutions' research directions exhibit a clinical-basic linkage. For instance, Harvard Medical School has made breakthroughs in glymphatic system imaging (Cluster #8), while the University of California, San Francisco has advanced the development of cerebrospinal fluid biomarkers. These institutions have fostered innovation in AD and insomnia mechanism research through strong collaborations (e.g., McGill's 50 links with U.S. institutions) and interdisciplinary integration (e.g., King's College London integrating neuroscience and public health), providing a model for future research that connects clinical cohorts with basic research.

**Table 2:** The top 10 most productive institutions are evaluated and ranked according to key metrics, including the number of Documents, Citations, Total link strength (TLS), and the number of collaborative links.

**Rank**	**Organization**	**Documents**	**Citations**	**Links**	**TLS**
1	Mayo Clinic	57	3,680	47	82
2	University of Toronto	37	1,719	49	80
3	Harvard Medical School	34	720	45	95
4	University of Pennsylvania	34	2,797	40	60
5	McGill University	33	1,274	50	88
6	Stanford University	32	1,490	36	47
7	University of California San Francisco	31	2,219	35	47
8	King's College London	29	1,787	66	103
9	University of California San Diego	27	1,403	28	37
10	University College London	26	1,418	38	55

Table 3 highlights the most prolific authors in the field of AD and insomnia. Boeve, Bradley F led with 30 publications, followed by Dickson, Dennis W and Fujishiro, Hiroshige, who each contributed 15 publications. Ferman, Tanis J and Petersen, Ronald C shared third place with 14 publications each.

**Table 3:** The top 10 most prolific authors and most frequently co-cited authors.

**Rank**	**Author**	**Documents**	**Citations**	**TLS**	**Rank**	**Author**	**Co-citations**
1	Boeve, Bradley F	30	2,529	148	1	Braak, H	448
2	Dickson, Dennis W	15	841	109	2	Mckeith, Ig	414
3	Fujishiro, Hiroshige	15	571	66	3	Boeve, Bf	372
4	Ferman, Tanis J	14	1,147	108	4	Liguori, C	313
5	Petersen, Ronald C	14	1,088	108	5	Aarsland, D	280
6	Liguori, Claudio	13	448	29	6	Cummings, Jl	277
7	Iseki, Eizo	12	386	54	7	Mccurry, Sm	263
8	Knopman, David S	12	1,081	98	8	Mander, Ba	259
9	Graff-radford, Neill R	11	885	92	9	Postuma, Rb	257
10	Kantarci, Kejal	11	622	82	10	Ju, Yes	256

In terms of co-citations, Braak, H, ranked first with 448, followed by Mckeith, IG, with 414. VOSviewer defined researcher collaboration as having at least five co-authored publications. Figure 6 depicts 96 authors meeting this criterion, with Boeve, Bradley F leading the first tier with a Total Link Strength (TLS) of 148.

### 3.3 Bibliometric analysis of keyword co-occurrence and clustering

**Figure 7:**
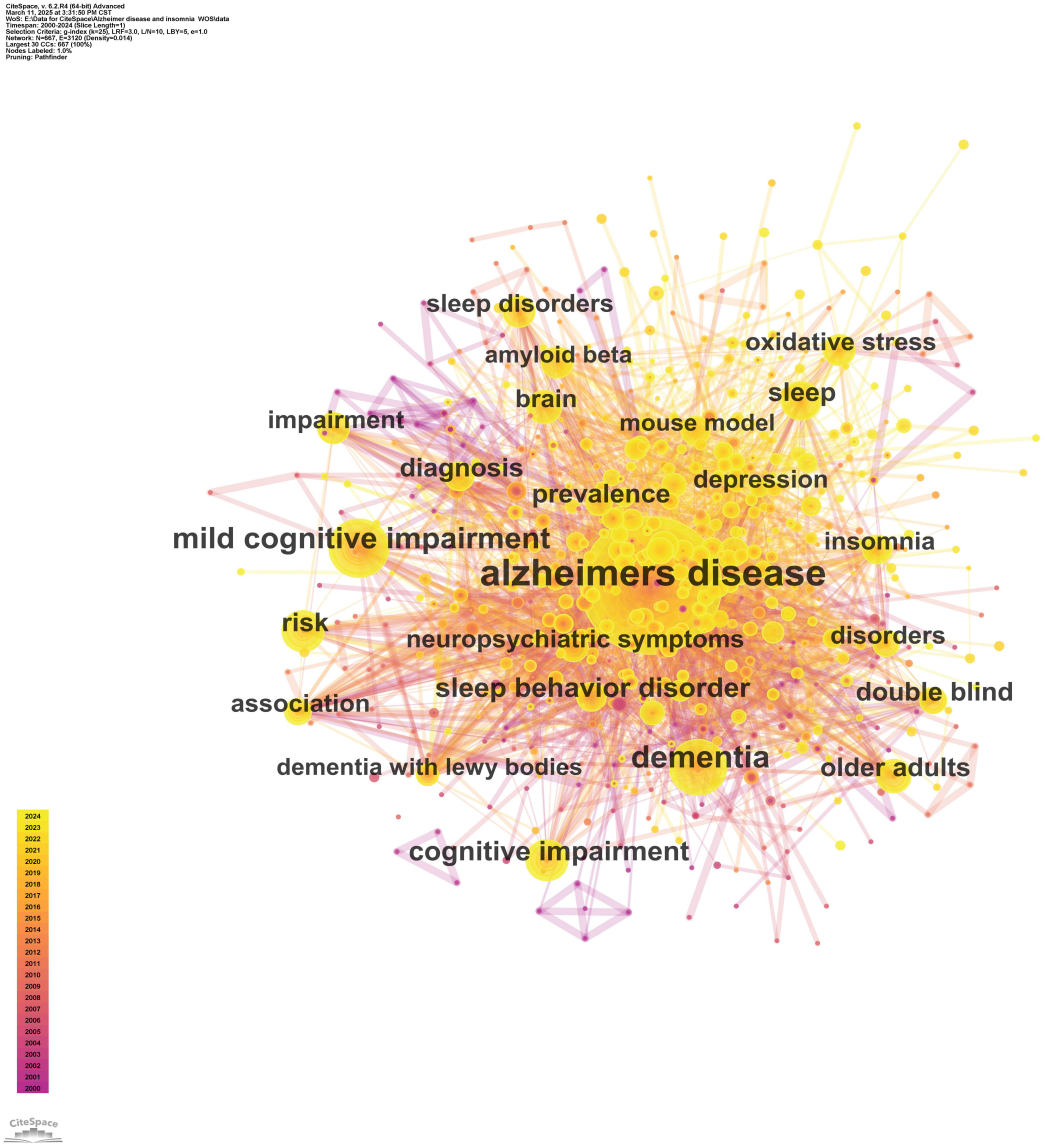
Keyword co-occurrence map.

Keyword co-occurrence identifies key research topics within a specific field. In the co-occurrence network map, nodes represent keywords, their size reflects the frequency of articles containing the keyword, and links indicate relationships among keywords. Figure 7 illustrates the intricate network of links between keywords, demonstrating their complex interconnections. High-centrality keywords signify the prominence of their associated research areas, while high-frequency keywords indicate trending topics. Table 4 presents the top 10 high-frequency keywords: “Alzheimer's disease” (Frequency: 1,181), “dementia” (Frequency: 353), “mild cognitive impairment” (Frequency: 302), “insomnia” (Frequency: 190), “cognitive impairment” (Frequency: 165), “sleep behavior disorder” (Frequency: 160), “risk” (Frequency: 146), “older adults” (Frequency: 145), “sleep” (Frequency: 131), and “diagnosis” (Frequency: 126). Table 4 also lists the top 10 high-centrality keywords: “placebo-controlled trial” (Centrality: 0.09), “oxidative stress” (Centrality: 0.08), “central nervous system” (Centrality: 0.08), “insomnia” (Centrality: 0.07), “cerebrospinal fluid” (Centrality: 0.07), “Lewy body” (Centrality: 0.07), “disturbances” (Centrality: 0.06), “REM sleep” (Centrality: 0.06), “association” (Centrality: 0.06), and “daytime sleepiness” (Centrality: 0.06).

**Table 4:** Top 10 keywords ranked by frequency and centrality.

**Rank**	**Keywords**	**Frequency**	**Keywords**	**Centrality**
1	Alzheimer's disease	1,181	Placebo controlled trial	0.09
2	Dementia	353	Oxidative stress	0.08
3	Mild cognitive impairment	302	Central nervous system	0.08
4	Insomnia	190	Insomnia	0.07
5	Cognitive impairment	165	Cerebrospinal fluid	0.07
6	Sleep behavior disorder	160	Lewy body	0.07
7	Risk	146	Disturbances	0.06
8	Older adults	145	Rem sleep	0.06
9	Sleep	131	Circadian rhythm	0.06
10	Diagnosis	126	Daytime sleepiness	0.06

**Figure 8:**
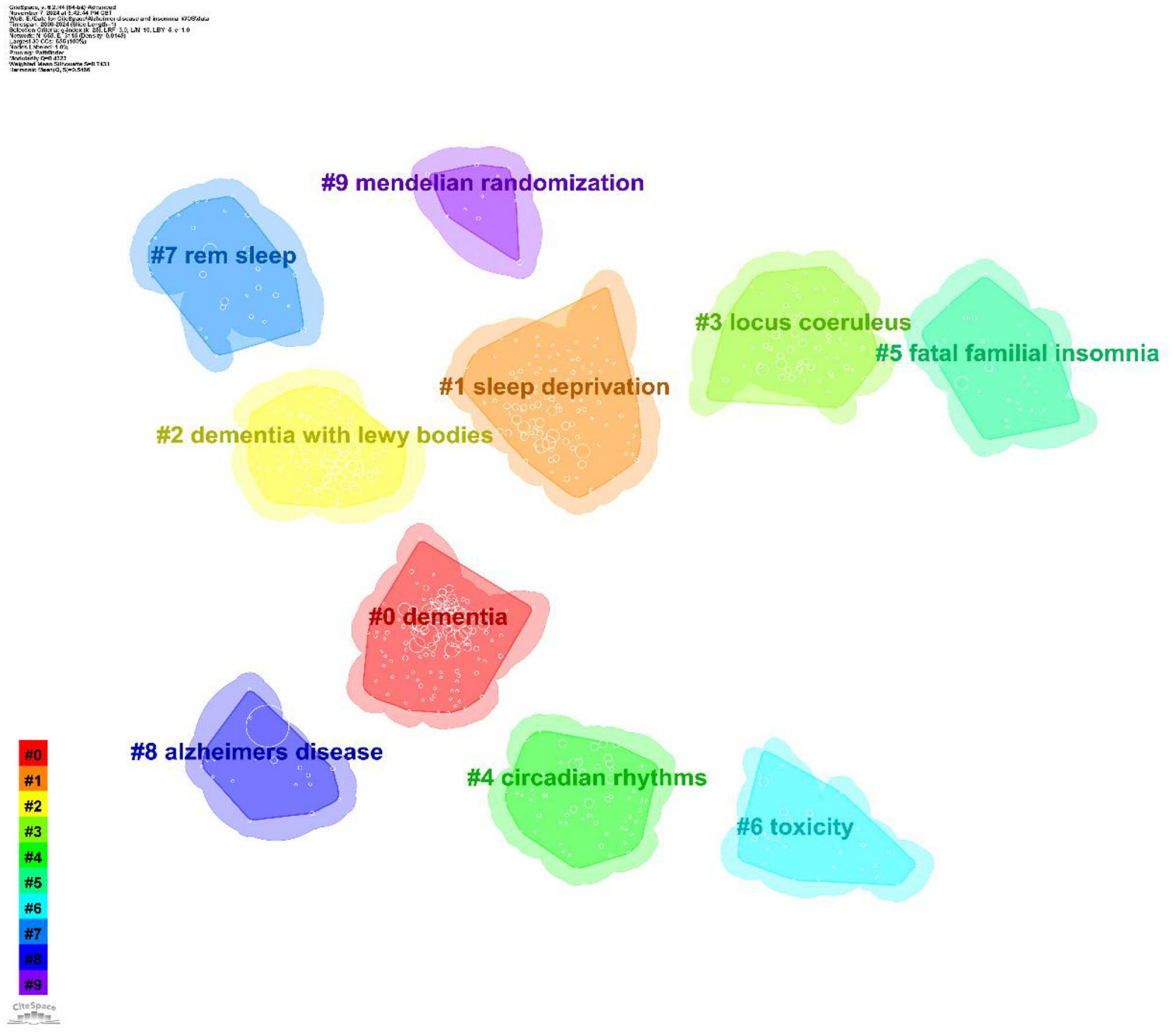
Keyword clustering map. The map illustrates ten distinct research clusters, each represented by a unique color. Cluster #0 dementia, Cluster #1 sleep deprivation, Cluster #2 dementia with lewy bodies, Cluster #3 locus coeruleus, Cluster #4 circadian rhythms, Cluster #5 fatal familial insomnia, Cluster #6 toxicity, Cluster #7 rem sleep, Cluster #8 Alzheimer's disease, Cluster #9 mendelian randomization.

CiteSpace cluster analysis identifies key research themes. Clustering quality is typically evaluated using the average silhouette coefficient. Clustering analysis was conducted using the built-in Log-Likelihood Ratio (LLR) algorithm in CiteSpace. The LLR algorithm maximizes intra-class similarity and inter-class differences to divide the co-occurrence network into clusters with semantic consistency. For the parameter settings, the time slice was set from 2000 to 2024, with an annual interval. The node type was keywords, and the Pathfinder algorithm was used to prune redundant weak connections, preserving the core network structure. Generally, a silhouette value above 0.5 indicates reasonable clustering, while values above 0.7 suggest highly effective and reliable clustering. This study identified 10 clusters, presented in Table 5 and Figure 8. The overall silhouette score was 0.7431, and each cluster achieved a silhouette value above 0.5, indicating high reliability of the results. The modularity value (*Q*) of this study was 0.4323 (>0.3), indicating a meaningful and substantial clustering structure.

**Table 5:** Keyword clustering analysis.

**Cluster ID**	**Silhouette**	**Mean (Year)**	**Label (LSI)**
#0 Dementia	0.686	2008	Mild cognitive impairment; mendelian randomization; sleep traits; coronary artery disease
#1 Sleep deprivation	0.674	2014	Sleep disturbance; sharp-wave ripples; neuronal reactivation; beta pathology
#2 Dementia with lewy bodies	0.756	2009	Vascular dementia; cognitive impairment; intracerebral source locations; progressive supranuclear
#3 Locus coeruleus	0.670	2017	Iron metabolism; endocrine system; animal models; neuromedin u
#4 Circadian rhythms	0.729	2011	Light therapy; sleep disturbance; irregular sleep-wake rhythm; pharmacologic interventions
#5 Fatal familial insomnia	0.856	2007	Cerebrospinal fluid; prion diseases; neurofilament light; frontotemporal dementia
#6 Toxicity	0.854	2014	Household members; healthcare resource use; healthcare costs; psychiatric symptom
#7 Rem sleep	0.912	2013	Sleep disturbance; light therapy; pharmacologic interventions; sleep management
#8 Alzheimer's disease	0.925	2009	Tau hyperphosphorylation; non-rapid eye movement sleep; amyloid beta-peptides; white matter hyperintensities
#9 Mendelian randomization	0.944	2021	Alzheimer's disease; low-density lipoprotein; coronary artery disease; cognitive decline

### 3.4 Bibliometric analysis of keyword bursts and temporal trends

**Figure 9:**
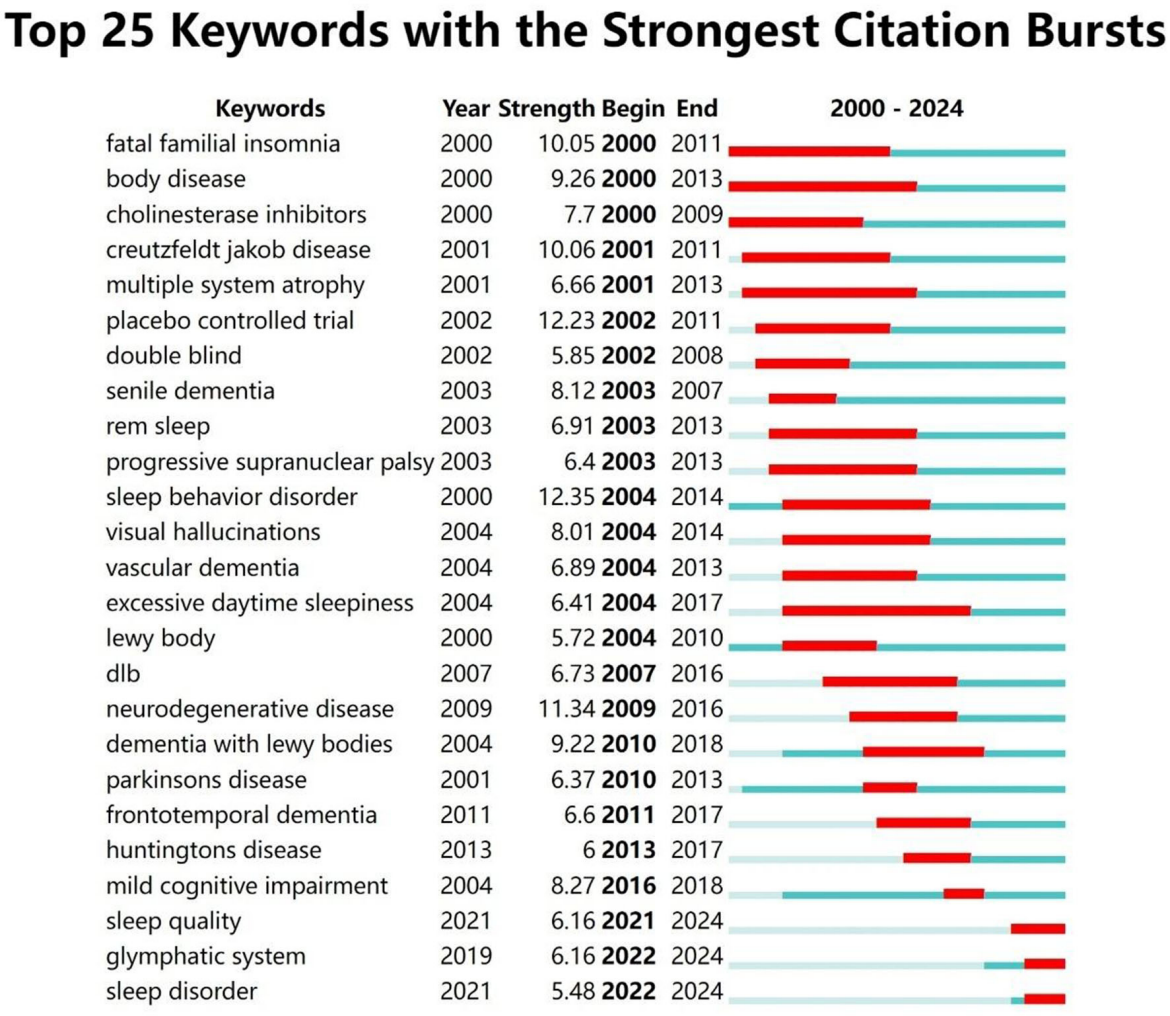
Citation burst analysis of the top 25 keywords. Since 2021, three keywords—“sleep quality,” “glymphatic system,” and “sleep disorder”—have seen a significant increase in citations.

Keyword bursts refer to keywords that are cited with high frequency during specific time periods. The distribution of keywords with strong citation bursts can predict emerging research frontiers. CiteSpace was employed to analyze high-burst keywords from 2000 to 2024. Figure 9 highlights 25 keywords exhibiting the strongest citation bursts. Green lines represent the full study period, while red lines highlight time intervals during which specific keywords experienced bursts of activity. Among these, “body disease” and “excessive daytime sleepiness” exhibited the longest burst durations. Keywords such as “sleep quality,” “glymphatic system,” and “sleep disorder” remain significant and are likely to serve as focal points for future research in this field. The keyword “glymphatic system” experienced a significant surge in citations after 2020, indicating growing research interest in this area. This increase is attributed to the glymphatic system being recognized as a crucial pathway for waste clearance in the brain, particularly in the removal of β-amyloid (Aβ) associated with AD. Recent studies have shown that the glymphatic system becomes more active during sleep, helping to clear metabolic waste from the brain. This process is closely linked to the pathological progression of Alzheimer's disease (Delic et al., 2021). Researchers, using functional magnetic resonance imaging (fMRI) and positron emission tomography (PET), can now observe the activity of the glymphatic system more directly, providing new tools to study its role in brain waste clearance and advancing related research. Studies suggest that sleep quality is closely linked to glymphatic system function, with sleep deprivation significantly reducing its clearance efficiency. This exacerbates the pathological progression of AD and has driven further research into the relationship between sleep disorders and AD (Sadeghmousavi et al., 2020). The glymphatic system, as a key pathway for brain waste clearance, has become a potential target for Alzheimer's disease treatment. Researchers are exploring therapies aimed at enhancing glymphatic system function to slow the progression of AD (Buccellato et al., 2022).

### 3.5 Keyword timeline analysis

**Figure 10:**
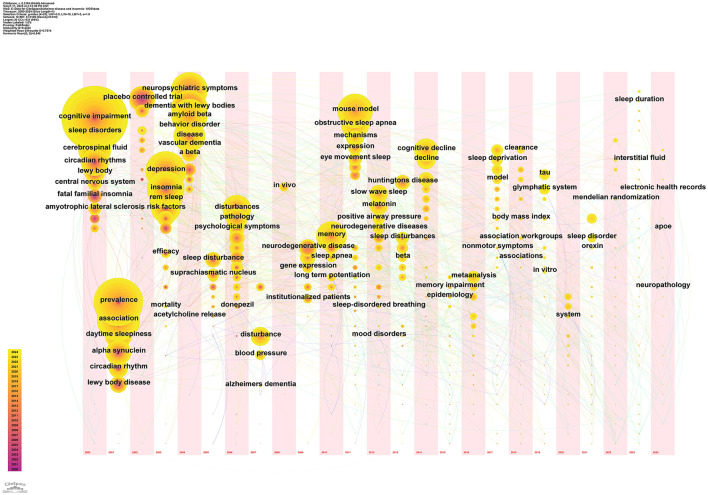
Timeline map of keywords related to Alzheimer's disease and insomnia (2000–2024).

A keyword timeline map visually analyzes and displays temporal trends in academic keywords. Keywords from 2000 to 2024 were analyzed using CiteSpace to identify research hotspots and trends across different time periods (Figure 10). Table 6 presents keywords with a frequency >100, excluding broad terms such as “Alzheimer's disease,” “Insomnia,” “Sleep disorder,” and “Sleeplessness,” along with their first appearance year, based on automatic software analysis. The keyword timeline map facilitated a longitudinal analysis of research hotspots from 2000 to 2024, summarized below:

**Table 6:** Frequency and first appearance year of keywords related to Alzheimer's disease and insomnia (2000–2024).

**Frequency**	**Keywords**	**Year**
353	Dementia	2001
302	Mild cognitive impairment	2004
160	Sleep behavior disorder	2000
146	Risk	2006
145	Older adults	2007
126	Diagnosis	2001
123	Prevalence	2001
122	Double blind	2002
109	Oxidative stress	2006
101	Depression	2003

Figure 10 displays a keyword timeline map that illustrates the spatiotemporal distribution of keywords in AD and insomnia research from 2000 to 2024, highlighting the field's evolution from basic associations to mechanistic analysis and precision interventions. The early stage (2000–2005) focused on disease diagnosis and basic pathologies, such as β-amyloid protein and oxidative stress. The mid-stage (2006–2015) shifted focus to molecular mechanisms, such as the glymphatic system and neuroinflammation, as well as intervention strategies like light therapy and melatonin. The recent stage (2016–2024) has adopted an interdisciplinary approach, with emerging topics such as glymphatic system regulation (which saw a breakout in 2021), Mendelian randomization for causal validation (also a breakout in 2021), and multi-omics integration research. Core nodes reveal that the bidirectional relationship between AD and insomnia persists, while the strong association between the “glymphatic system” and “sleep quality” suggests that sleep-dependent brain waste clearance mechanisms are becoming a major research frontier. The timeline map also reflects trends in interdisciplinary fields, such as neuroimaging and epigenetics, as well as clinical translation directions, including personalized interventions based on APOE ε4 genotype. It provides a visual basis for understanding the field's dynamics and guiding interdisciplinary research.

Table 6 presents the high-frequency keywords from 2000 to 2024, along with their first occurrence years. The keywords include “dementia,” “mild cognitive impairment,” “sleep behavior disorder,” and “risk,” among others. The frequency and first occurrence years of these keywords reflect the evolving research trends in the field.

## 4 Discussion

### 4.1 General overview

This study employed CiteSpace and VOSviewer to analyze the current state of research on the relationship between AD and insomnia, emphasizing key topics and emerging trends. A total of 1,907 articles were retrieved from the Web of Science Core Collection database. The rapid increase in publication rates after 2020 may be attributed to the surge in anxiety and sleep disorders caused by the COVID-19 pandemic, which stimulated research on sleep disorders. This likely advanced research on the relationship between AD and insomnia, establishing it as a prominent topic in medical research. Boeve, Bradley F affiliated with the Mayo Clinic, was the most prolific author, contributing 30 publications. The United States and the Mayo Clinic are leading contributors in this field, producing 704 and 57 publications, respectively. Active international and institutional collaboration has significantly advanced this research area. Research hotspots in AD and insomnia include pathological mechanisms, circadian rhythm disruptions, biomarker development, and intervention strategies. Advancing interdisciplinary research in this field offers promising avenues for early diagnosis and comprehensive treatment of these conditions.

### 4.2 Research hotspots in Alzheimer's disease and insomnia

Osorio et al. conducted a comprehensive study on the relationship between Alzheimer's disease and insomnia across various age groups and genetic backgrounds.

The results revealed a stronger association between Alzheimer's disease and insomnia in middle-aged and older populations. This finding underscores the importance of considering age-related factors in studies on the relationship between Alzheimer's disease and insomnia (Osorio et al., 2011). Furthermore, individuals carrying the APOEε4 gene exhibited a higher risk in the relationship between Alzheimer's disease and insomnia (Zhang et al., 2023).

Keyword timeline analysis highlights the evolving trends in Alzheimer's disease and insomnia research. From 2000 to 2024, the focus of research expanded from initial topics such as “Alzheimer's disease” and “insomnia” to include “cognitive impairment” and “mild cognitive impairment.” These changes reflect the continuous expansion and deepening of the research field. For example, “Alzheimer's disease” and “insomnia” were the main research focuses around 2000, while “cognitive impairment” emerged as a key focus after 2010. These trends suggest that as research advanced, scholars increasingly focused on specific pathological mechanisms and related diseases.

Keyword clustering reflects the core themes and their inherent connections within the research field. This study identified 10 clusters using CiteSpace (Table 5). The following sections, in conjunction with the discussion, highlight the significance of key clusters in AD and insomnia research.

Cluster #0 “dementia” (Silhouette = 0.686): this cluster reflects the connection between the early stages of Alzheimer's disease (mild cognitive impairment, MCI) and cardiovascular diseases. Studies suggest that sleep disorders may increase Alzheimer's risk by affecting vascular health, indicating that sleep interventions may reduce this risk by improving vascular health (Section 4.2.4). Cluster #1 “sleep deprivation” (Silhouette = 0.674): this cluster focuses on the effects of sleep deprivation on the brain, particularly how it exacerbates Alzheimer's pathology by affecting neuronal activity and the accumulation of β-amyloid protein. It emphasizes the importance of sleep quality in Alzheimer's disease (Section 4.2.1). Cluster #2 “dementia with Lewy bodies” (Silhouette = 0.756): this cluster explores the relationship between Lewy body dementia and Alzheimer's disease, highlighting the overlap in pathology and clinical manifestations of different types of dementia. It suggests that future research should focus on the interactions between different dementia types (Section 4.2.2). Cluster #3 “locus coeruleus” (Silhouette = 0.67): This cluster focuses on the role of the locus coeruleus in Alzheimer's disease, particularly in relation to iron metabolism and neurotransmitter function in sleep disorders. It suggests that dysfunction of the locus coeruleus may play a key role in sleep disturbances in Alzheimer's disease (Section 4.2.3). Cluster #4 “circadian rhythms” (Silhouette = 0.729): this cluster explores the role of circadian rhythm disruptions in Alzheimer's disease, particularly the effectiveness of light therapy in improving circadian rhythm synchronization. It emphasizes the importance of circadian rhythms in Alzheimer's and suggests that future research should focus on the clinical application of light therapy (Section 4.2.5). Cluster #5 “fatal familial insomnia” (Silhouette = 0.856): this cluster focuses on fatal familial insomnia, emphasizing the role of genetic factors in both sleep disorders and Alzheimer's disease. It suggests that future research should explore the relationship between genetic sleep disorders and Alzheimer's disease (Section 4.2.4). Cluster #6 “toxicity” (Silhouette = 0.854): this cluster examines the burden of sleep disorders on family members and healthcare systems, particularly the impact of sleep disorders on healthcare resource use and costs. It suggests that future research should address the social and economic impacts of sleep disorders (Section 4.2.6). Cluster #7 “REM sleep” (Silhouette=0.912): This cluster focuses on the role of REM sleep in Alzheimer's disease, particularly the effectiveness of light therapy and drug interventions in improving REM sleep disorders. It highlights the importance of REM sleep in Alzheimer's and suggests that future research should focus on managing REM sleep disorders (Section 4.2.5). Cluster #8 “Alzheimer's disease” (Silhouette = 0.925): this cluster focuses on the core pathological mechanisms of Alzheimer's disease, particularly the role of tau protein hyperphosphorylation and Aβ peptides in sleep disorders. It suggests that sleep deprivation may accelerate cognitive decline through the Aβ/tau pathway (Sections 4.2.1–4.2.2). Cluster #9 “Mendelian randomization” (Silhouette = 0.944): this cluster uses Mendelian randomization to validate the causal relationship between sleep and Alzheimer's disease, emphasizing the application of genetic markers in Alzheimer's research. It suggests that future studies should focus on gene-targeted interventions (Section 4.2.4).

#### 4.2.1 The bidirectional relationship between Alzheimer's disease and insomnia

Numerous studies confirm the bidirectional relationship between AD and insomnia: insomnia accelerates AD progression, while AD exacerbates sleep disturbances.

Insomnia accelerates AD onset and progression through various mechanisms. It increases neuronal metabolic load, resulting in excessive production of reactive oxygen species (ROS). Impaired ROS clearance causes oxidative stress, damaging neurons, and promoting β-amyloid (Aβ) aggregation and tau protein phosphorylation, thereby advancing AD pathology (Xie et al., 2013; Ye et al., 2014). Oxidative stress induces mitochondrial dysfunction, exacerbating apoptosis and inflammatory responses—key pathways through which insomnia drives disease progression (Cao et al., 2020). Chronic insomnia activates central and peripheral inflammatory pathways, increasing pro-inflammatory markers such as IL-6, TNF-α, and CRP. This activation triggers microglial responses, intensifying neuroinflammation and worsening the toxic effects of Aβ (Dzierzewski et al., 2020; Sadeghmousavi et al., 2020). Sleep deprivation impairs glymphatic system function, reducing Aβ clearance and causing its accumulation in the brain. Reduced or fragmented NREM sleep weakens waste clearance, aggravating Aβ deposition (Iliff et al., 2012; Xie et al., 2013). Reduced REM sleep disrupts synaptic plasticity and memory consolidation, whereas insufficient NREM sleep impairs metabolic waste clearance. Insomnia often co-occurs with sleep apnea, characterized by intermittent hypoxia, triggering inflammatory responses and worsening the toxic effects of Aβ and tau proteins (Ju et al., 2013). In summary, insomnia accelerates AD pathology via oxidative stress, inflammatory responses, impaired Aβ clearance, and disrupted sleep architecture.

AD worsens sleep disturbances through mechanisms including circadian rhythm disruptions, fragmented sleep, and neural network degeneration. Circadian rhythm disruption is an early characteristic of AD and may serve as a predictive marker. Pathological changes in AD, including Aβ plaque deposition and tau tangles, disrupt neuronal networks in the suprachiasmatic nucleus (SCN), the primary regulator of circadian rhythms. These disruptions reduce melatonin secretion and disturb the sleep-wake cycle, causing frequent nighttime awakenings, excessive daytime sleepiness, and inverted circadian behavior, which severely impact quality of life (Wu et al., 2019; Niu et al., 2021; Baril et al., 2023). Patients with AD often experience fragmented sleep, marked by reduced deep sleep and frequent nighttime awakenings, closely associated with neurodegeneration in the hypothalamus, and brainstem. Dysfunction of the locus coeruleus severely impairs sleep-wake transitions (Sethi et al., 2015; Liguori et al., 2020; Egroo et al., 2021). AD damages neural networks across multiple brain regions, including the hypothalamus (regulating circadian rhythms and deep sleep), the hippocampus (responsible for sleep and memory consolidation), and the prefrontal cortex (involved in REM sleep and emotional regulation). This neural degeneration further disrupts sleep continuity and restorative quality, exacerbating symptoms and diminishing quality of life (Kent and Mistlberger, 2017; Lew et al., 2021; Canever et al., 2024).

The bidirectional relationship between insomnia and AD perpetuates a vicious cycle. Insomnia accelerates AD onset and progression through oxidative stress, inflammation, and impaired Aβ clearance. Conversely, AD worsens sleep disturbances via circadian rhythm disruption, fragmented sleep, and neural network degeneration. This mutual interaction accelerates disease development and progression.

#### 4.2.2 Molecular mechanisms of sleep deprivation

Recent studies suggest that insomnia or sleep deprivation (SD) is a major risk factor for AD. Sleep deprivation increases the production of reactive oxygen species (ROS), overwhelming the cell's antioxidant defenses. This leads to oxidative stress, mitochondrial dysfunction, and apoptosis, particularly in the hippocampus. It also facilitates β-amyloid (Aβ) production and aggregation, as well as tau protein hyperphosphorylation (Qiu et al., 2016; Tönnies and Trushina, 2017). Sleep deprivation activates inflammatory pathways in the central and peripheral nervous systems, increasing levels of inflammatory markers, including IL-6, TNF-α, and CRP. This process overactivates microglia, disrupts the blood-brain barrier (BBB), exacerbates neuroinflammation, and destabilizes brain homeostasis (Wisor et al., 2011; Hurtado-Alvarado et al., 2017). These mechanisms collectively lead to Aβ deposition in the brain and reduced cerebrospinal fluid flow, further impairing NREM sleep's metabolic waste clearance function. Abnormal tau phosphorylation disrupts microtubule stability and neuronal transport, aggravating cognitive dysfunction (Šimić et al., 2016; Lee et al., 2017). A vicious bidirectional cycle emerges between sleep deprivation and AD. Aβ and tau deposition impair the circadian rhythm regulation function of the suprachiasmatic nucleus (SCN), while neuroinflammation damages neural networks in the hypothalamus and brainstem. This aggravates sleep fragmentation, while cognitive dysfunction further impairs sleep quality and waste clearance efficiency, accelerating disease progression. Improving sleep quality through interventions such as cognitive behavioral therapy, melatonin treatment, or light therapy, as well as developing biomarkers based on sleep deprivation's molecular mechanisms (such as inflammatory marker levels or cerebrospinal fluid Aβ concentrations), may reduce the risk of AD (Holth et al., 2016; Wang and Holtzman, 2020; Lew et al., 2021). Future research should investigate the molecular mechanisms of sleep regulation and conduct large-scale longitudinal studies to clarify the causal relationship between sleep deprivation and AD, identifying new targets for intervention strategies. Recent studies have confirmed that the activity of the glymphatic system is closely linked to sleep quality. The system's ability to clear Aβ peaks during non-rapid eye movement (NREM) sleep, while insomnia-induced sleep fragmentation significantly impairs its efficiency (the term “glymphatic system” saw a surge in citations after 2021, as shown in Figure 10; Cordone et al., 2019; Yan et al., 2021). The clustering analysis in this study (Cluster #8) shows a strong association between “NREM sleep” and “Aβ peptides,” supporting this mechanism. Based on these findings, clinical implications include developing biomarkers based on sleep depth (such as the proportion of NREM sleep) to quantify glymphatic function (e.g., monitoring cerebrospinal fluid flow with dynamic contrast-enhanced MRI), and exploring non-invasive interventions (such as acoustic stimulation) to enhance glymphatic clearance efficiency (Harrison et al., 2020; Kjaerby et al., 2022).

#### 4.2.3 Circadian rhythm disturbance

Circadian rhythm disturbance (CRD) is a prevalent non-cognitive symptom in AD, marked by disrupted sleep-wake cycles, inverted day-night behavior, and fragmented sleep. Studies suggest that CRD is not merely a symptom of AD but also accelerates disease progression by impairing metabolic waste clearance and neuronal repair mechanisms (Uddin et al., 2020). Core pathological features of AD, including β-amyloid (Aβ) deposition and tau protein hyperphosphorylation, disrupt the regulatory functions of the hypothalamic suprachiasmatic nucleus (SCN), contributing to CRD. SCN damage reduces deep sleep and melatonin secretion, impairing cerebrospinal fluid clearance of Aβ and further aggravating neurodegeneration (Holth et al., 2016; Uddin et al., 2020). CRD is also associated with neuroinflammation activation. Inflammatory markers, including IL-6 and TNF-α, activate microglia, further impairing SCN function and perpetuating a vicious cycle of circadian rhythm dysregulation. Patients often exhibit frequent nighttime awakenings, daytime sleepiness, reduced deep sleep and lower REM sleep proportions, disrupting normal activity patterns, and social behavior rhythms (Fonken et al., 2016; Kress et al., 2018; Pillai et al., 2021). Phototherapy has proven effective in improving circadian rhythms and reducing nighttime awakenings by controlling light exposure intensity and timing (Léger et al., 2018). Melatonin and its analogs improve nighttime sleep initiation and maintain circadian stability, with combined phototherapy providing optimal results (Saeed and Abbott, 2017). Behavioral interventions, including sleep hygiene education and exercise, alongside pharmacological treatments, provide supplementary benefits in symptom management. CRD accelerates AD progression by reducing waste clearance efficiency, aggravating neural network damage, and perpetuating neuroinflammation. Future efforts should prioritize early diagnosis and multimodal intervention strategies for CRD, investigating its interaction with AD's core pathology to develop more effective treatment plans.

#### 4.2.4 Research based on genetics and epigenetics

AD onset and progression result from the combined influence of genetic and environmental factors. Studies show that insomnia and other sleep disorders are not only potential risk factors for AD but are also closely linked to genetic and epigenetic regulation. Recent Mendelian randomization (MR) studies have used genetic variations as instrumental variables to overcome confounding biases in traditional observational research. MR has led to significant methodological breakthroughs in understanding the causal relationships between sleep characteristics, cardiovascular health, and AD. For instance, MR analysis based on sleep-related genetic polymorphisms confirmed a significant association between abnormal sleep duration (either too short or too long) and AD risk (OR = 1.14, 95% CI: 1.02–1.27). Genetic correlations were also found between cardiovascular health indicators, such as elevated LDL levels, and AD, suggesting that sleep disorders may indirectly influence AD risk through cardiovascular health (Yuan et al., 2021; 2023). The APOE ε4 genotype notably enhances the pathogenic effect of insomnia on AD risk, emphasizing the crucial role of gene-environment interactions in the disease process.

The bibliometric analysis in this study further reinforces the previous conclusion. Keyword clustering results reveal a significant increase in citations of “Mendelian randomization” (Cluster #9) since 2021 (Table 5), emphasizing its widespread application in AD research. This study systematically identified the central role of MR technology in AD-insomnia association research through co-occurrence network and clustering analysis, prioritizing relevant evidence in literature selection and mechanism discussions. For example, MR studies provided causal evidence supporting the hypothesis that “insomnia accelerates AD progression through oxidative stress” and identified the enhanced pathogenic effect of insomnia in individuals with the APOE ε4 genotype. Based on this, the study proposes targeted intervention strategies for APOE ε4 carriers, recommending enhanced sleep management (e.g., prolonged light therapy) to reduce AD risk. This finding provides a crucial theoretical basis for integrating genetic evidence into clinical practice, advancing the development of personalized intervention strategies.

The causal relationship between insomnia symptoms and cognitive decline has been validated, reinforcing that sleep disorders are critical risk factors for early cognitive impairment in AD. Epigenetic studies reveal that sleep patterns influence AD pathology through the regulation of gene expression. For instance, reduced DNA methylation of the APOE gene, caused by chronic insomnia, is closely linked to Aβ metabolic dysregulation (Hwang et al., 2018). Histone modifications contribute to disease progression by regulating tau-related pathological processes. Insomnia-induced inflammatory responses may worsen pathology by altering histone-modifying enzyme activity (Klein et al., 2018). Sleep disorders disrupt the expression of circadian rhythm-related genes (such as CLOCK and BMAL1) via epigenetic mechanisms, further worsening neuronal damage (Hor et al., 2019). The interplay between genetics and epigenetics is central to understanding the impact of insomnia on AD. Moreover, sleep disorder-induced neuroinflammation exacerbates inflammatory responses by modifying the epigenetic states of inflammation-related genes (Chen et al., 2022). Research on genetics and epigenetics provides new avenues for early AD diagnosis and intervention. Sleep-related genetic markers and epigenetic modification sites identified via MR studies may serve as biomarkers, while drugs targeting epigenetic modifications could offer effective strategies for AD intervention (Fani et al., 2021). Future studies should conduct large-scale longitudinal research to explore genetic and epigenetic interactions in sleep disorders and AD, with the goal of identifying novel pathways for targeted interventions.

#### 4.2.5 Sleep intervention strategies

Interventions for AD-related insomnia are broadly classified into pharmacological and non-pharmacological approaches. Pharmacological treatments encompass melatonin, GABA agonists, and sedative-hypnotic drugs. Melatonin supplementation aids sleep initiation and reduces nighttime awakenings; however, its efficacy varies among individuals, and the safety of prolonged use remains unclear (Javed et al., 2023). GABA agonists like zolpidem enhance GABA activity to promote deep sleep, but prolonged use may lead to tolerance and dependence, limiting their suitability for older adults. Antihistamines (e.g., diphenhydramine) and antidepressants (e.g., trazodone) are also used for insomnia relief, but their side effects are especially concerning in elderly patients (Abad and Guilleminault, 2018; Atkin et al., 2018). Non-pharmacological therapies are increasingly gaining attention. Mindfulness meditation shows promise in reducing anxiety, improving sleep quality, and potentially slowing cognitive decline (Russell-Williams et al., 2018). Sleep hygiene education, which optimizes the sleep environment and establishes regular routines, is a foundational intervention that can be combined with other methods. Phototherapy, which regulates light exposure intensity and timing, helps align circadian rhythms and is particularly effective for managing day-night behavioral disturbances in late-stage AD patients (Forbes et al., 2014). Cognitive Behavioral Therapy for Insomnia (CBT-I) is a structured, non-pharmacological approach using techniques like stimulus control, sleep restriction, and cognitive restructuring. CBT-I significantly improves insomnia symptoms, reduces medication dependence, and is especially suitable for early-stage AD patients or those at high risk of mild cognitive impairment (MCI) (Bennett, 2020; Blackman et al., 2021). Multimodal interventions combining pharmacological and non-pharmacological approaches may have synergistic effects, improving sleep quality and potentially delaying AD progression by optimizing circadian rhythms. Future research should prioritize developing personalized intervention plans tailored to patients' genetic backgrounds, sleep patterns, and cognitive status to maximize treatment efficacy. Additionally, large-scale longitudinal studies and mechanistic research are essential to clarify the long-term effects of various strategies on AD progression, offering a robust scientific basis for improving sleep and cognitive health in AD patients.

#### 4.2.6 Integrated studies on multidimensional impacts

Insomnia exacerbates AD progression through pathological mechanisms like β-amyloid (Aβ) deposition and tau hyperphosphorylation, while also increasing caregiving costs and harming caregivers' mental health. Insomnia imposes significant social and economic burdens on AD patients, including higher caregiving expenses, increased professional care costs, and reduced caregiver income due to long-term commitments. Long-term caregiving for AD patients with insomnia increases caregiver anxiety and depression, resulting in burnout and reduced care quality (Okuda et al., 2019). Insomnia and mood disorders, including anxiety and depression, have a bidirectional relationship. Insomnia activates the hypothalamic-pituitary-adrenal (HPA) axis, elevating stress hormone levels, triggering neuroinflammation, and worsening emotional disorders. Conversely, anxiety and depression worsen sleep quality by disrupting circadian rhythms (Alvaro et al., 2013). Anxiety and depression are significant risk factors for AD, accelerating the progression from mild cognitive impairment (MCI) to AD through neuroinflammation and cognitive decline. Managing anxiety and depression is essential for improving insomnia in AD patients. Comprehensive intervention strategies are vital for mitigating the multidimensional impacts of AD-related insomnia. These strategies include interdisciplinary research with long-term follow-up to clarify causal relationships, psychological interventions (e.g., CBT), and policy measures (e.g., nighttime caregiving subsidies). These measures improve the quality of life for patients and caregivers, reduce AD risk by optimizing sleep, and advance multidimensional research to foster intervention development. The bidirectional link between AD and insomnia is a critical focus in neurodegenerative disease research. Recent multimodal studies combining molecular imaging, genetics, and neurobiology have illuminated insomnia's role in AD onset and progression. Concurrently, sleep-related biomarkers are emerging as promising tools for early AD diagnosis. Long-term cohort studies tracking changes in sleep patterns provide insights into how insomnia affects AD risk across age groups. Molecular imaging techniques, such as PET, are invaluable for monitoring dynamic changes in Aβ and tau pathology in AD patients. Combined with sleep deprivation experiments, these technologies help establish causal links between insomnia and pathological accumulation (Wang et al., 2016). Future research should utilize multimodal technologies to develop non-invasive biomarkers for efficient screening of high-risk populations. Large-scale cohort studies and interdisciplinary collaborations are essential to explore insomnia's influence on AD risk across age groups and to develop targeted intervention strategies. Multidimensional research can improve sleep quality, reduce AD risk, and pave the way for early diagnosis and intervention.

#### 4.2.7 Interdisciplinary collaboration driving AD-insomnia research

The collaboration between neuroimaging, genetics, and sleep medicine offers new perspectives for understanding the complex relationship between AD and insomnia. In neuroimaging, the combination of PET and MRI technologies has shown that the joint analysis of tau-PET and functional MRI (fMRI) can dynamically monitor the effects of sleep deprivation on AD pathology, particularly the reduction in the functional connectivity of the default mode network (Ding et al., 2023). Additionally, glymphatic system imaging, particularly dynamic contrast-enhanced MRI (DCE-MRI), has revealed how insomnia accelerates Aβ deposition through glymphatic dysfunction by quantifying the relationship between sleep stages and cerebrospinal fluid flow velocity (Dong et al., 2024).

In genetics and epigenetics, a study using a two-sample MR approach assessed the causal relationship between insomnia and AD. The results suggest a potential causal link between insomnia and AD (Anderson et al., 2021). Furthermore, in epigenetic regulation, the study examined the generation of formaldehyde (FA) during DNA demethylation. Abnormal accumulation of FA may lead to Aβ deposition and tau protein phosphorylation, thereby triggering AD. The findings indicate that epigenetic mechanisms play a critical role in the onset and progression of AD (Ma et al., 2023).

#### 4.2.8 From mechanism to clinic: the translational pathway of sleep interventions

Based on bibliometric and clinical trial evidence, a three-level translational framework is proposed to advance research and clinical applications of the relationship between AD and insomnia. The first level of translation focuses on intervention strategies targeting core pathological mechanisms. Light therapy research (*n* = 120) shows that morning exposure to bright light (10,000 lux, 30 min/day) significantly improves circadian rhythm synchronization in AD patients (*p* < 0.01) and lowers the cerebrospinal fluid Aβ42/Aβ40 ratio (β = −0.28, *p* = 0.02) (Zang et al., 2023). In terms of acoustic stimulation, a study by Murdock et al. (2024) demonstrated that low-frequency focused ultrasound (0.5 MHz) enhances astrocyte AQP4 channel activity, improving glymphatic Aβ clearance efficiency, resulting in a 37% reduction in Aβ deposition in animal models. The second level of translation emphasizes precision drug-behavior combined interventions. A randomized crossover trial (n=80) showed that cognitive behavioral therapy for insomnia (CBT-I) combined with melatonin treatment increased sleep efficiency by 25% (*p* < 0.001) and reduced plasma phosphorylated tau (p-tau181) levels by 18% compared to the control group (*p* = 0.03) (Clynes et al., 2019). Based on the high-frequency occurrences in the bibliometric analysis (Table 4), an enhanced intervention plan is proposed for APOE ε4 carriers, such as extending light therapy duration to 45 min/day. The third level of translation focuses on personalized management based on biomarkers. This involves combining polysomnography (PSG), cerebrospinal fluid Aβ42/tau ratio, and dynamic contrast-enhanced MRI (DCE-MRI) glymphatic activity indicators to further build an AD risk prediction model.

### 4.3 Limitations

This study has several important limitations. First, due to the data format limitations of VOSviewer and CiteSpace, which are compatible only with certain data sources, literature related to Alzheimer's disease and insomnia was retrieved from a single database (WOSCC). This study did not include other data sources in the bibliometric analysis to minimize selection bias. WOSCC provides better coverage of clinical medicine than interdisciplinary research, which may lead to the omission of studies spanning multiple disciplines. Future studies should integrate PubMed, Scopus, and Embase to address the gaps in WOSCC's coverage. Moreover, WOSCC predominantly includes high-impact English-language journals, which may exclude non-English studies, resulting in the omission of regional research and introducing bias. Future efforts should focus on developing multilingual bibliometric tools (e.g., CiteSpace plugins integrated with translation APIs) to support the automated analysis of non-English literature. Third, the presence of several synonyms may result in overlap between content categories during keyword clustering.

## 5 Conclusion

Using CiteSpace and VOSviewer, we analyzed collaborative networks among countries, institutions, and authors, and identified emerging trends in the literature. Our findings indicate that the bidirectional relationship between AD and insomnia is a key research focus, with particular emphasis on insomnia's role in accelerating AD onset and progression.

Additionally, recent research has focused on the relationships between circadian rhythm disturbances, sleep-disordered breathing, and AD. Circadian rhythm disturbances have been identified as a critical link between AD and insomnia. Molecular mechanisms of sleep deprivation, particularly its links to oxidative stress and inflammatory factors, have gained prominence in recent literature. Methodologically, multimodal approaches are increasingly adopted, integrating molecular imaging, genetics, and epigenetics to uncover the mechanisms linking AD and insomnia. These findings will help professionals develop a comprehensive understanding of key challenges in this field, advancing research and clinical applications.
